# Potential biomarkers for predicting the risk of thyroid cancer in immunosenescence: a population-based and externally validated multi omics study

**DOI:** 10.3389/fonc.2024.1525767

**Published:** 2025-01-21

**Authors:** Qian Li, Yuanyuan Zhao, Jiawei Yan, Chao He

**Affiliations:** ^1^ Department of Emergency Medicine, Yongchuan Hospital, Chongqing Medical University Affiliated Hospital, Chongqing, China; ^2^ Department of Orthopaedics, Yongchuan Hospital, Chongqing Medical University Affiliated Hospital, Chongqing, China

**Keywords:** DNA methylation hannum age, generated genetic risk scores, meta-analysis, thyroid cancer, external validation

## Abstract

**Objectives:**

Genome-wide association studies (GWAS) have pinpointed several risk loci linked to thyroid cancer; however, the discovery of new plasma proteins implicated in immunosenescence continues to pose significant challenges. This study aims to uncover novel plasma proteins tied to aging, potentially contributing to thyroid cancer, utilizing diverse investigative methodologies.

**Methods:**

In this research, we utilized an integrative omics approach to identify novel plasma proteins associated with immunosenescence in relation to the risk of thyroid cancer. Additionally, we performed meta-analyses to pinpoint loci and genes affected by pleiotropic effects. Finally, complementary results were obtained from an independent cohort analyzed at Chongqing Medical University Yongchuan Hospital and Bulk-RNA seq from GEO database.

**Results:**

Causal analysis suggests that DNA methylation age acceleration as measured by the Hannum method increases the risk of thyroid cancer (OR: 1.126, 95% CI: 1.002-1.265, P=0.046). Subsequently, we conducted a meta-analysis on the relationship between Hannum DNA methylation age and thyroid cancer risk, which identified 138 potential risk loci through FUMA. Additionally, proteomics and transcriptomics collectively identified 6 potential targets related to immunosenescence and thyroid cancer. Subsequently, Bulk-seq results indicated differential expression of *GFRA2* and *LILRA2* genes in thyroid cancer. Finally, analyses from an independent cohort at the Second Affiliated Hospital of Chongqing Medical University also demonstrated high expression of *LILRA2* in thyroid cancer patients.

**Conclusions:**

This study identified novel plasma proteins associated with immunosenescence that may be linked to thyroid cancer development. These findings enhance our understanding of the immunosenescence-thyroid cancer link and support future diagnostic and therapeutic developments.

## Introduction

Thyroid cancer represents the most prevalent form of endocrine malignancy, with a global annual incidence of 15.7 cases per 100,000 people (1). Each year, approximately 567,233 new cases are diagnosed, and 41,071 fatalities are reported worldwide (2, 3). This type of cancer is subdivided into four main categories based on pathogenesis, histopathological characteristics, and clinical manifestations: differentiated, poorly differentiated, anaplastic, and medullary thyroid cancers. Of these, differentiated thyroid cancers are the most common, constituting about 80% of all cases and generally associated with a favorable prognosis (4). Given the substantial economic impact thyroid cancer imposes on patients (5), early detection and effective management are essential to mitigate its detrimental effects. Although traditional diagnostic methods are widely employed in clinical practice, their sensitivity remains suboptimal. In this context, plasma proteins have garnered significant attention as potential diagnostic biomarkers. As functional molecules within the body, plasma proteins typically exhibit alterations during disease onset and progression, particularly in the early stages of cancer. Consequently, plasma proteins hold promise as crucial tools for the early screening and diagnosis of thyroid cancer.

Aging, a complex and multifactorial process, leads to a generalized functional decline across all bodily organs and tissues. The question of whether aging stems from a single causal mechanism or multiple origins remains unresolved (6). Epigenetic clocks serve as precise aging markers, correlating with various diseases, including thyroid cancer, through the use of weighted linear combinations of CpGs and DNA methylation to estimate chronological age (7). Increasingly, research underscores a robust link between aging and the development of thyroid cancer, with studies also connecting DNA methylation to the aging process (8). Perhaps due to the important role metabolism plays in inflammation and aging (9–11). Therefore, examining the role of DNA methylation in thyroid cancer is crucial. While there is growing evidence that accelerated aging is linked to age-related diseases and conditions, some investigations report no association between epigenetic clocks and thyroid cancer (12).

Recent progress in transcriptomic and proteomic profiling has dramatically enhanced our ability to access gene products specific to tissues and circulating systems, relevant to both health and disease scenarios. However, linking gene transcript or protein abundance directly to phenotypes in observational studies may be prone to confounding or could indicate reverse causation (13). Integrating genetic data with transcripts, proteins, and phenotypic information enables the identification of distinct biomarkers as potential causal agents in diseases (14). For instance, Gusev et al. utilized single-nucleotide polymorphisms (SNPs) in cis to genetically infer transcripts and associate them with GWAS summary statistics for selected traits, thereby demonstrating causality via a transcriptome-wide association study (TWAS) (15). We have recently adapted this approach for proteome-wide association studies (PWAS), applying genetically imputed plasma proteome models integrated with transcriptomic data to explore potential therapeutic targets for thyroid cancer (16). Employing TWAS and PWAS as instrumental variable (IV) analyses, akin to two-sample MR, these genetic models are limited to the cis-regions of gene transcripts or proteins, helping to reduce confounding risks like horizontal pleiotropy through a focus on nearby genetic variants (17). Additionally, colocalization analyses address multiple causal variants in the same region, thus diminishing the potential for genetic confounding arising from linkage disequilibrium (18–20). In brief, in this study, we utilized an integrative omics approach that combines genomic, transcriptomic, and proteomic data to investigate the role of immune senescence in thyroid cancer risk. Initially, we conducted GWAS to identify genetic loci associated with thyroid cancer, providing a foundation for exploring the potential pleiotropic effects of aging on cancer susceptibility. We then applied Mendelian randomization to assess the impact of DNA methylation age acceleration, gaining insights into the epigenetic processes of aging and their potential influence on cancer risk. To further explore the molecular mechanisms, proteomics was used to identify plasma proteins differentially expressed due to immune senescence, shedding light on aging-related molecular changes and their role in tumor progression. Additionally, Bulk-RNA sequencing was utilized to examine gene expression changes in thyroid cancer tissues, revealing genes associated with aging and immune senescence. By integrating genetic, epigenetic, proteomic, and transcriptomic data, we developed a comprehensive framework to understand how aging and immune senescence influence thyroid cancer. This approach facilitates the identification of potential biomarkers for early detection and therapeutic targets, offering new opportunities to improve diagnosis and treatment strategies.

**Figure 1:**
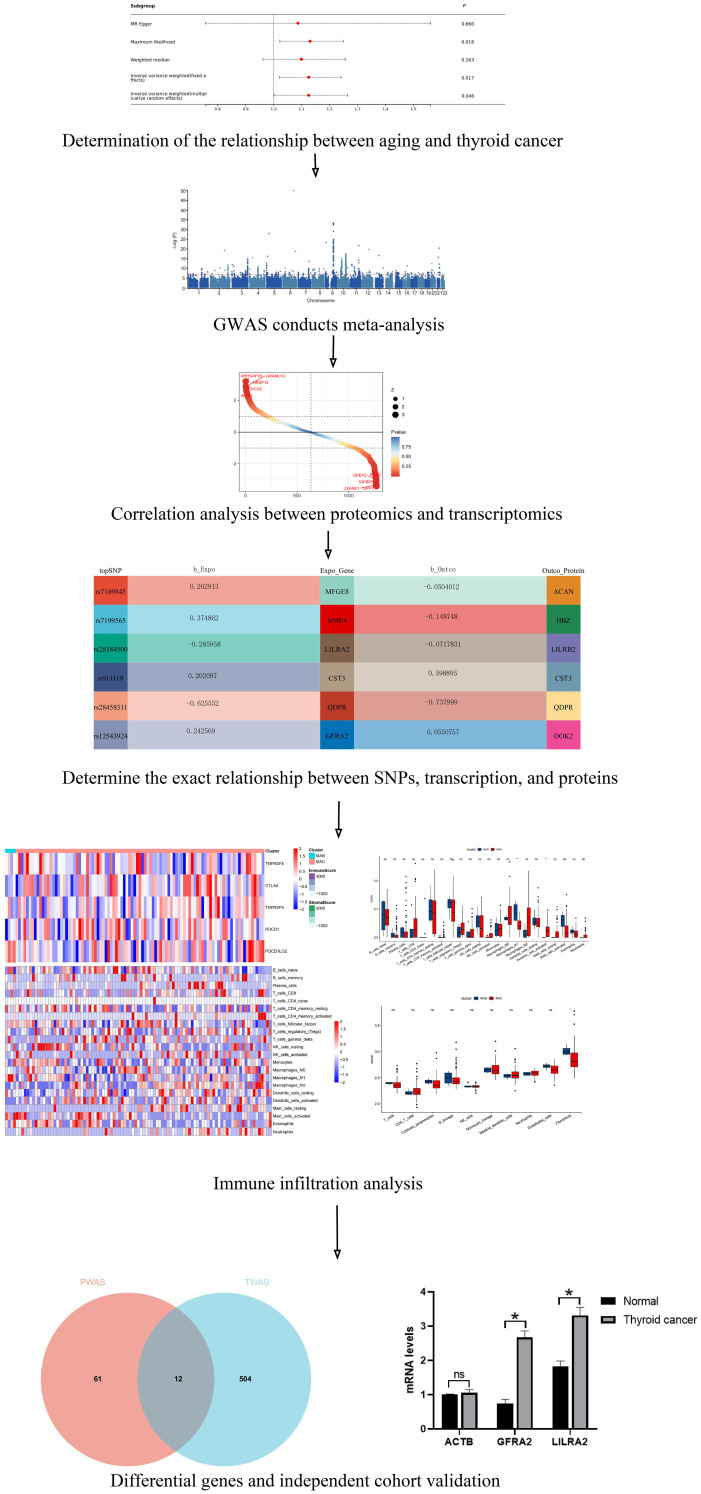
The flowchart systematically described our study. ns indicates no significance, *P<0.05.

In our research, we executed a detailed omics pipeline analysis aimed at pinpointing potential drug targets linking aging and thyroid cancer. The methodology is outlined in **Figure 1**. Initially, we applied TWAS and PWAS to ascertain potential causal plasmas for aging and thyroid cancer, leveraging GWAS data. To corroborate our results, we performed external validation using an independent cohort from Chongqing Medical University Yongchuan Hospital, supplemented with Bulk-RNA sequencing data from the GEO database.

## Materials and methods

### Date source

The study design is shown in **Figure 1**. Genetic variants associated with the aging process were identified from an extensive meta-analysis in the GWAS Catalog. This analysis encompassed 34,710 individuals from 28 European cohorts, focusing on DNA methylation age acceleration measures including Hannum age acceleration (34,449 samples, 7,541,726 SNPs), PhenoAge acceleration (34,463 samples, 7,545,555 SNPs), and GrimAge acceleration (34,467 samples, 7,544,493 SNPs) (7). Similarly, genetic data pertaining to thyroid cancer were obtained from the same GWAS Catalog, derived from a large-scale prospective cohort study comprising over 459,000 European individuals (21). The gene expression matrix for thyroid cancer patients used in this study was sourced from the Gene Expression Omnibus (GSE27155), a database maintained by the National Center for Biotechnology Information.

### Selection of instrumental variables

Initially, SNPs were filtered using Plink Software based on the following criteria: a P-value less than 5×10⁻⁸, a genetic distance of 10,000 kb, and a linkage disequilibrium parameter (r²) less than 0.01 from the GWAS focused on the aging process. Further, both the catalog and PhenoScanner databases were employed to determine if these SNPs had associations with known confounders, such as epigenetic age acceleration. Any SNP identified as linked to these confounders was excluded from our study (22).

### Statistical analysis

In this study, the primary technique for deducing causal relationships was the inverse variance weighted (IVW) method. This method was implemented using fixed effects models in scenarios without horizontal multiplicity heterogeneity, and random effects models when such heterogeneity was evident. To enhance the robustness and validate the IVW results, several supplementary methodologies were employed, including MR-Egger, MR-PRESSO, the weighted median approach, and maximum likelihood estimation. Specifically, maximum likelihood estimation refines the estimation of causal effects by optimizing the likelihood function under the assumption of a linear relationship between the exposure and the outcome. Conversely, the MR-Egger method undertakes weighted linear regression on the exposure outcomes, adhering to the InSIDE assumption, which necessitates rigorous scrutiny of the instrument variables’ validity. Furthermore, the MR-PRESSO method plays a crucial role in identifying and rectifying horizontal pleiotropy, ensuring that the IVW estimates remain unbiased from pleiotropic influences. Collectively, these methods establish a comprehensive analytical framework, significantly bolstering the study’s ability to accurately establish causal connections (23–25).

### Genetics risk score

To provide a thorough assessment of the association between alleles influencing exposure and the outcome, MR analyses were conducted bidirectionally using a weighted Genetic Risk Score (GRS) as IVs (26). This approach leveraged the same summary data as outlined previously in the study.

### Pleiotropy and heterogeneity analysis

In this investigation, heterogeneity was assessed using Cochrane’s Q statistic derived from the IVW method, with a p-value less than 0.05 indicating statistical significance. For identifying directional pleiotropy, both the MR-Egger intercept and MR-PRESSO methods were utilized, considering a p-value below 0.05 as indicative of the presence of pleiotropy. Specifically, the MR-PRESSO method is tailored to address pleiotropy through a structured three-step procedure: detection of horizontal pleiotropy, correction by removing outlier SNPs, and evaluation of causal estimates pre- and post-correction to ascertain any substantial changes. Additionally, the robustness of our results was further validated using a leave-one-out approach, which gauges the impact of individual SNPs on the overall Mendelian Randomization analysis. This approach helps confirm that the causal inferences are not unduly influenced by any single SNP, thereby enhancing the reliability of the findings.

### GWAS meta-analysis

To identify shared risk SNPs between aging and thyroid cancer, we conducted two cross-trait meta-analyses. The first was a GWAS meta-analysis performed using the Meta-analysis software. We then annotated the SNPs using ANNOVA and eqtl tools, and their potential risk loci were identified using FUMA, which helps pinpoint significant genetic associations and potential functional implications of the identified SNPs.

### Proteome-wide association studies

We utilized the FUSION tool to carry out PWAS (27). FUSION assessed the influence of SNPs on protein levels through various predictive models, including top1, blup, lasso, enet, and bslmm. The model exhibiting the highest predictive accuracy was selected for subsequent analysis. For this chosen model, we calculated protein weights specific to tissues relevant to the Meta-GWAS. These weights were then integrated with the genetic effects, represented by GWAS z-scores, to perform the PWAS. This integration involved computing the linear sum of the products of z-scores and weights for the independent SNPs at each locus.

### Transcriptome−wide association studies

We conducted a TWAS using the FUSION workflow, which incorporated tissue-specific weights relevant to Meta-GWAS (27). Our predictive models employed a range of methodologies. These models were aligned with LD reference data from the European ancestry cohort of the 1000 Genomes Project. This approach enabled a comprehensive analysis of how gene expression impacts Meta-GWAS phenotypes, facilitating a deeper understanding of genetic contributions to these traits.

### Summary-data-based Mendelian randomization

To explore the impact of SNPs on the expression of potential drug targets and their role in the development of Meta-GWAS phenotypes, we employed Summary-data-based Mendelian Randomization (SMR) analysis. This technique combines summary statistics from GWAS with data from protein Quantitative Trait Loci (pQTL) and expression Quantitative Trait Loci (eQTL) within a Mendelian Randomization framework, allowing for the assessment of associations between gene expression levels and target phenotypes. We focused our SMR analysis on genes relevant to them that showed significant SNP-heritability enrichment. In this context, genome-wide significant SNPs were used as instrumental variables. To determine whether the observed associations were attributable to linkage rather than pleiotropy or direct causality, we utilized the Heterogeneity In Dependent Instruments (HEIDI) test. Additionally, the HEIDI-outlier test, a component of the SMR approach, was specifically applied to distinguish between causality and pleiotropy by analyzing the homogeneity of effects across multiple loci.

### Evaluation of immune infiltration

In this study, many algorithms were used to calculate the immune infiltration (28).

### Differentially expressed genes

Differentially expressed genes (DEGs) were used with ‘wilcox’ test, limma analysis, t-test (29–31).

### Quantitative reverse−transcription polymerase chain reaction

In this study, we included three individuals diagnosed with thyroid cancer and three healthy subjects from Chongqing Medical University Yongchuan Hospital to undergo qPCR analysis (32, 33). We processed the collected blood by centrifuging it at 3,000 rpm for 10 minutes. After centrifugation, the supernatant serum was collected for mRNA extraction. Total RNA was extracted from the serum using the Trizol reagent (Life Technologies, USA). The extracted RNA was then converted to cDNA using the RevertAid First Strand cDNA Synthesis Kit (Fermentas, Canada). Amplification via PCR was conducted using the QuantiTect SYBR Green PCR Kit (Qiagen, Inc) on the ABI Prism 7000 detection system from Applied Biosystem (CA, USA). PCR amplification was carried out utilizing the QuantiTect SYBR Green PCR Kit (Qiagen, Inc) on the ABI Prism 7000 sequence detection system (Applied Biosystem, CA, USA). All experiments were conducted five times to ensure accuracy, and the specific primer sequences utilized are detailed in **Table 1**.

**Table 1:** RT-qPCR primer sequences.

Genes	Forward	Sequences
ACTB	Forward Primer	CATGTACGTTGCTATCCAGGC
	Reverse Primer	CTCCTTAATGTCACGCACGAT
GFRA2	Forward Primer	GGGCTCTTATGCTGGCATGAT
	Reverse Primer	AGTCCCTGAGGAACTTCTCAC
LILRA2	Forward Primer	CACTCATCAGAGTACAGTGACCC
	Reverse Primer	GTTCGAGTCATAAGCATAGCACC

## Results

### Mendelian randomization statistical of DNA methylation accelerations and thyroid cancer

**Figure 2:**
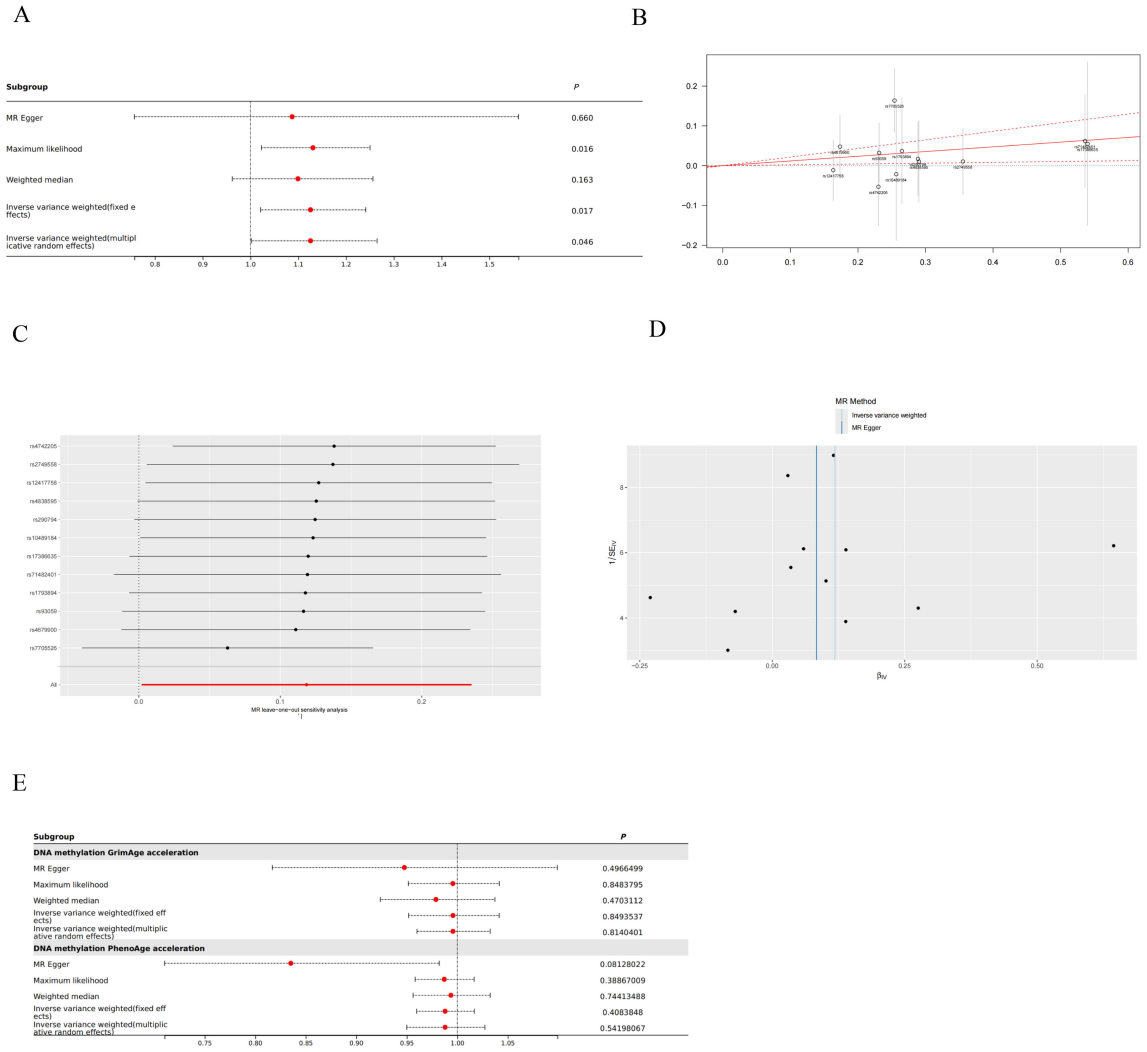
Mendelian randomization (MR) result in this study. **(A)** Forest plot for DNA Methylation Hannum age Accelerations and thyroid cancer. **(B)** Genetic Risk Score (GRS) plot for DNA Methylation Hannum age Accelerations and thyroid cancer. **(C)** Leave-one-out analysis for DNA Methylation Hannum age Accelerations and thyroid cancer. **(D)** Funnel plot for DNA Methylation Hannum age Accelerations and thyroid cancer. **(E)** Other forest plots for DNA methylation and thyroid cancer.

With IVW approach, we observed DNA methylation Hannum age acceleration correlated positively with thyroid cancer (OR:1.126, 95%CI: 1.002-1.265, P=0.046) (**Figure 2**). And the GRS results also suggest that DNA methylation Hannum age acceleration may be associated with the risk of thyroid cancer (Beta: 0.1185, SE: 0.049, P=0.017) (**Figure 2**). Additionally, pleiotropy and heterogeneity analysis reported no significant in this study. The MR-PRESSO results also indicated the absence of horizontal pleiotropy (*P*=0.159). This suggests that our analysis is robust and credible (**Figure 2**). Besides, funnel plot also confirmed that the causality between DNA Methylation Hannum age Accelerations and thyroid cancer was essentially unaffected by potential bias (**Figure 2**). Additionally, we did not find any significant correlation between other DNA Methylation Accelerations and thyroid cancer in relation to the thyroid (**Figure 2**).

### GWAS meta-analysis

**Figure 3:**
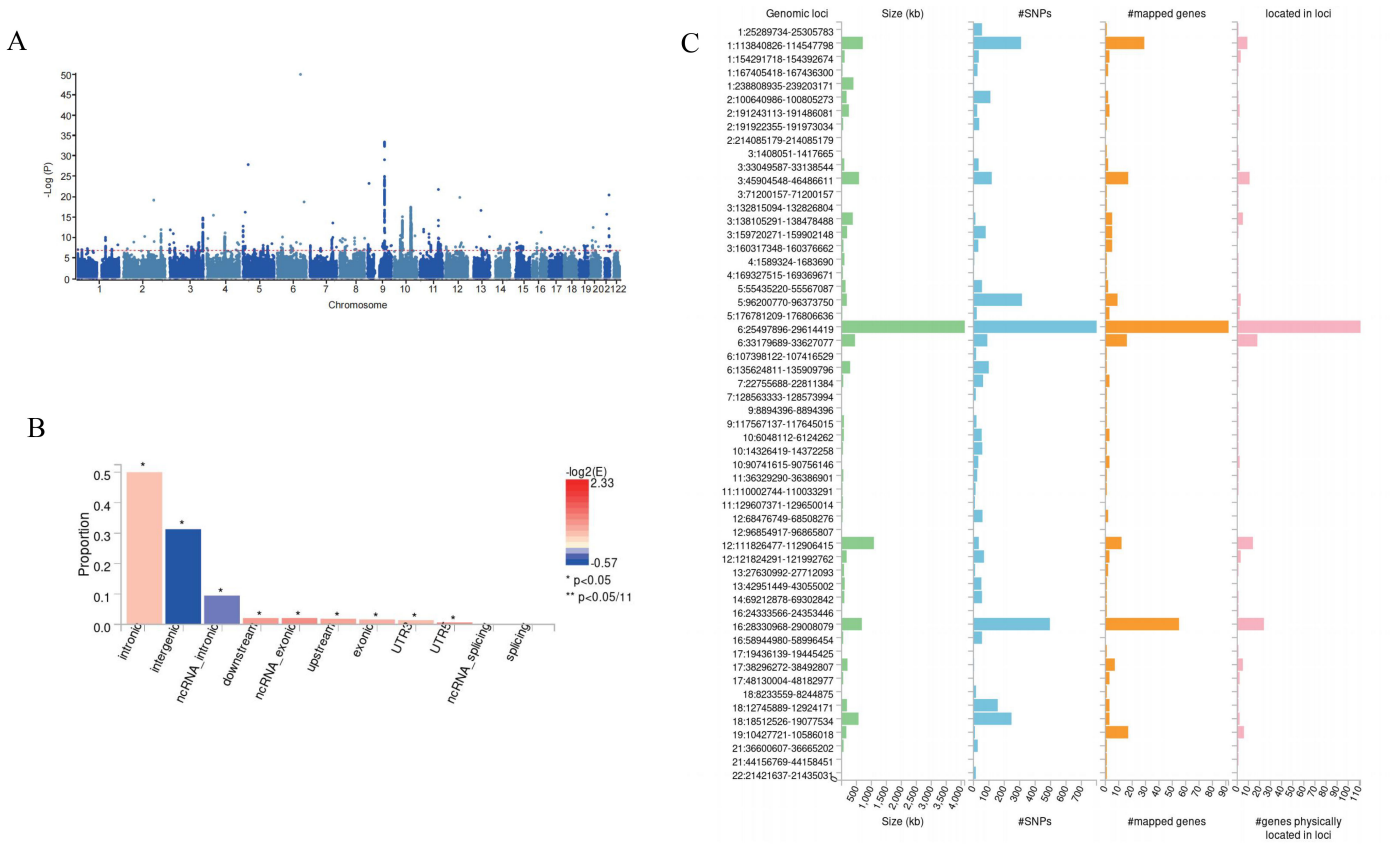
Meta-GWAS result in this study. **(A)** Manhattan plot for Meta-GWAS. **(B)** Potential risk loci from Meta-GWAS. **(C)** ANNOVA annotates the function of SNPs.

Subsequently, we conducted a meta-analysis of DNA methylation Hannum age and thyroid cancer (**Figure 3**), and FUMA identified a total of 138 potential risk sites (**Figure 3**, **Supplementary Table S1**). Subsequently, ANNOVA annotation results showed that most SNPs were located in the intron region (**Figure 3**, **Supplementary Table S2**). Finally, eqtl also showed that 131 genes are involved in DNA methylation Hannum age, promoting the occurrence of thyroid cancer (**Supplementary Table S3**).

### Search and validation of potential drug targets

**Figure 4:**
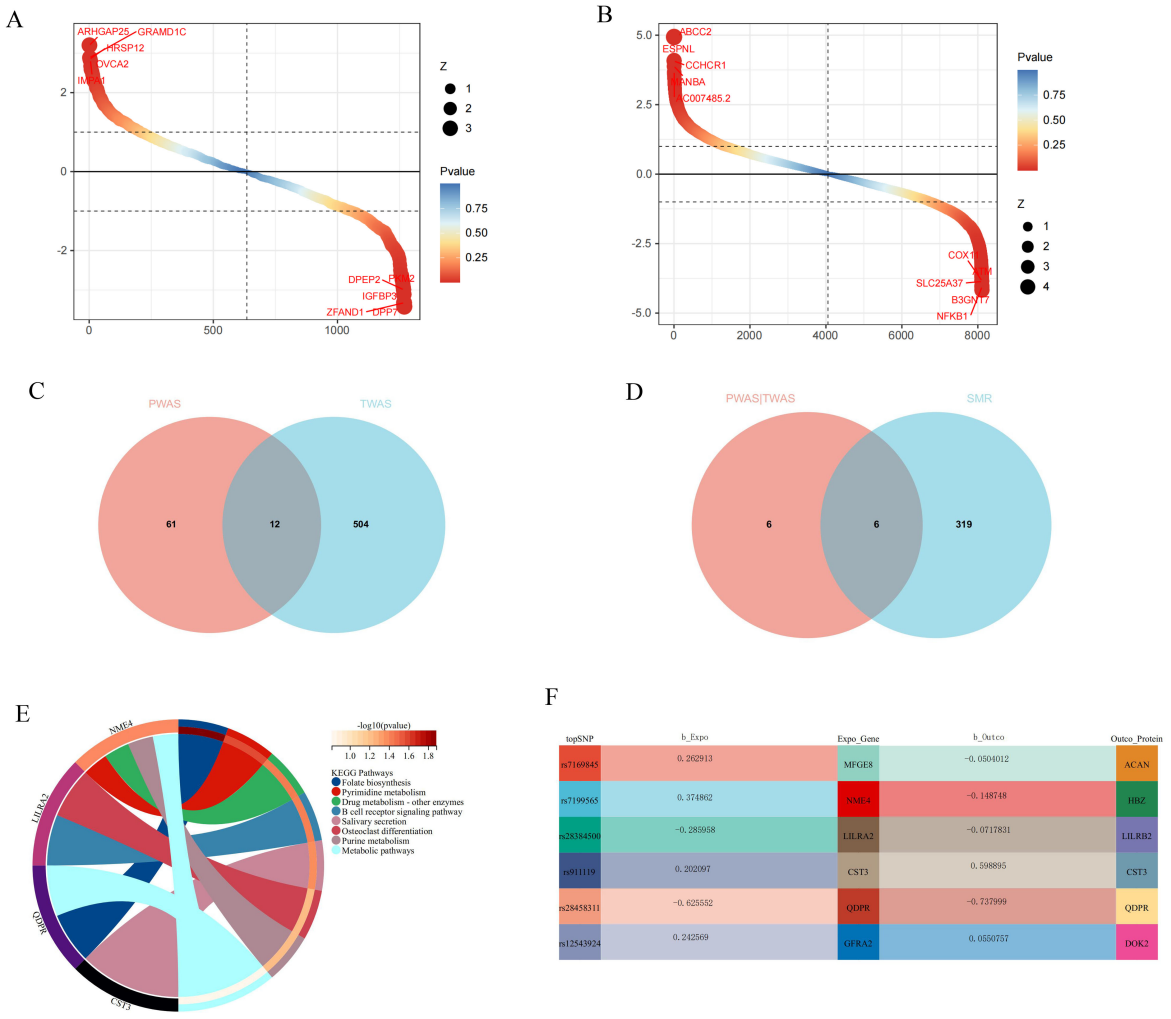
The results of the joint analysis of multiple omics in this study. **(A)** PWAS result in this study. **(B)** TWAS result in this study. **(C)** VNN result between PWAS and TWAS. **(D)** VNN result between PWAS and TWAS and SMR. **(E)** KEGG analysis of candidate genes. **(F)** The relationship between SNP regulated genes and proteins.

PWAS analysis revealed a close association between 76 proteins and GWAS-meta in ARIC (**Figure 4**). In TWAS, 516 genes were also closely associated with GWAS-meta (**Figure 4**). To determine whether SNPs affect GWAS-meta through the modulation of gene and protein expression, we employed SMR. The smr results found that the SNP’s influence on 6 genes could be due to causal relationships rather than pleiotropy (**Figure 4**).

### Association between the thyroid cancer and immune infiltration

**Figure 5:**
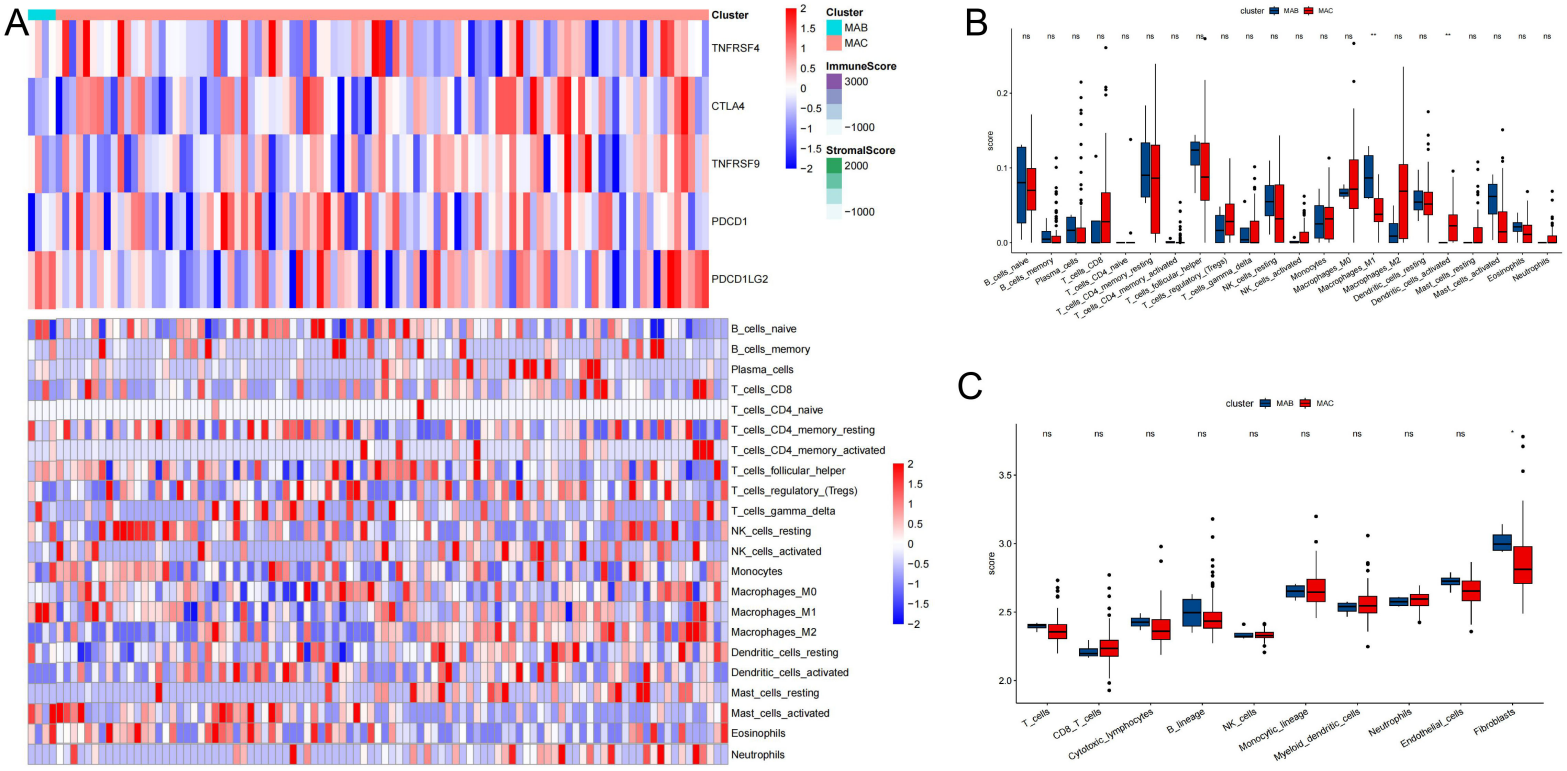
Association between thyroid cancer and immune infiltration. **(A)** Heatmap describing the immune infiltration landscape. **(B)** Boxplots describing the distribution of expression for the immune. **(C)** TME cells signatures (ns indicates no significance, *P < 0.05, **P < 0.01.

To delve deeper into immune infiltration and characterize the immune landscape, we quantified the abundance of 22 types of immune cells in the microenvironment, as depicted in **Figure 5**. In thyroid cancer patients, we observed differential expression levels across several immune cell subgroups, including M2 macrophages, neutrophils, CD8 T cells, dendritic cells, fibroblasts, among others. This analysis provided insights into the unique immune profiles associated with thyroid cancer, highlighting specific cellular dynamics that may influence disease progression and therapeutic responses.

### Expression levels of hub genes

**Figure 6:**
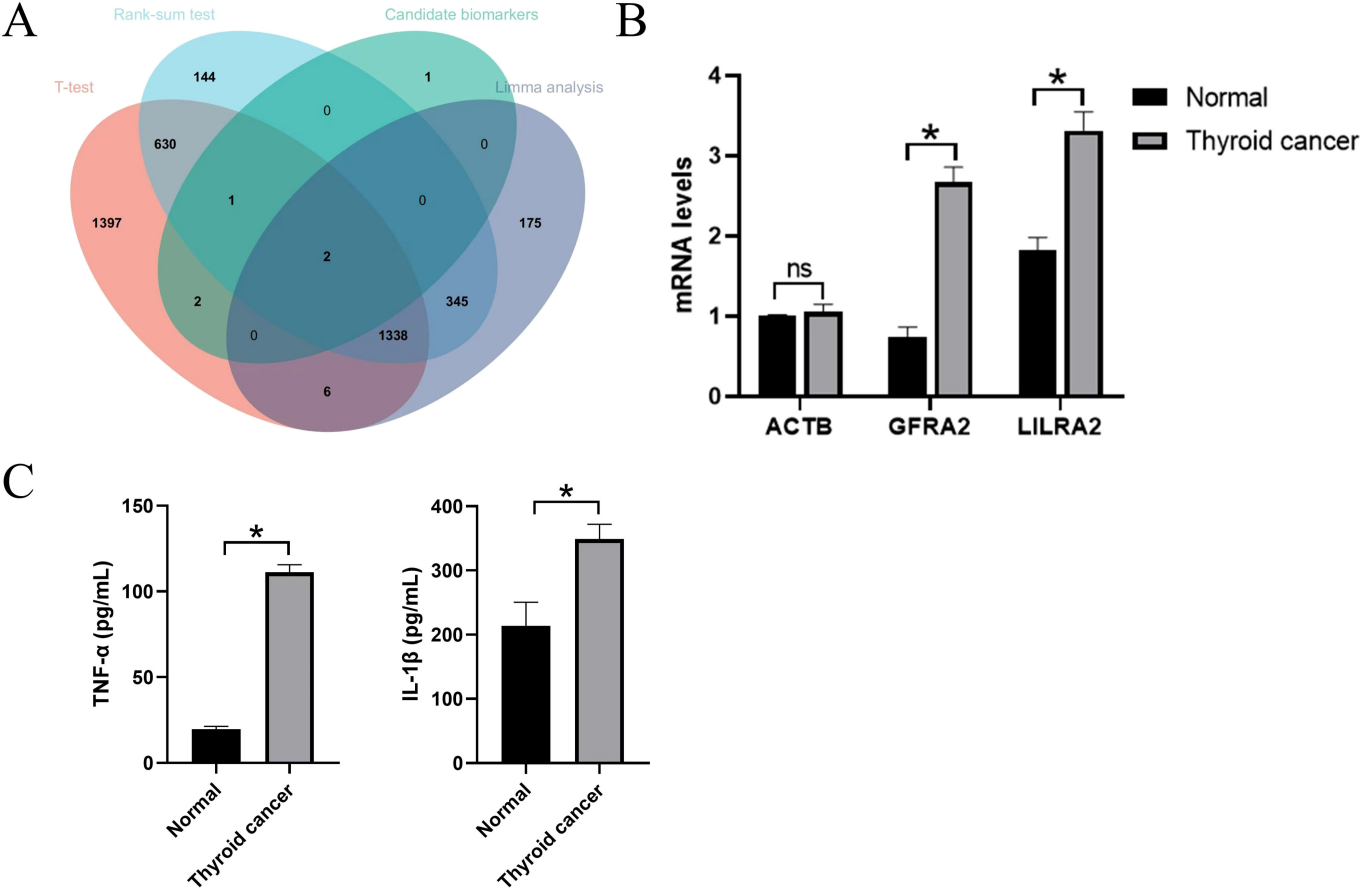
External validation of candidate genes. **(A)** Intersection with candidate genes under three different differential analysis methods. **(B)** QPCR results in candidate genes. ns indicates no significance, *P<0.05.

In DEG analysis, significant changes were observed in the expression of *GFRA2* and *LILRA2* genes in patients with thyroid cancer. When comparing serum mRNA expression levels between individuals with thyroid cancer and healthy controls, β-actin levels were found to be similar across both groups. However, there was a notable increase in the mRNA levels of *LILRA2* in the plasma of patients with thyroid cancer, with this difference reaching statistical significance (*P*<0.05). This suggests that an upsurge in transcriptome levels may influence the development of the nervous system, as illustrated in **Figure 6**.

## Discussion

In this study, we analysis using summary statistics from the GWAS Catalog to investigate the potential causal relationship between aging—specifically, epigenetic age acceleration as measured by DNA methylation Hannum age—and the risk of developing thyroid cancer. This investigation marks the first effort to assess how aging, quantified through changes in DNA methylation, may impact the likelihood of thyroid cancer onset. In addition, we further validated the above conclusion using an external queue to further demonstrate the relationship between the two.

To establish causal relationships from MR studies, it is crucial to assess biases stemming from potential violations of MR assumptions and to compare outcomes with observational studies to ensure the coherence of results. Thus, we compared our MR results with previous observational study outcomes to assess their reliability. Some studies reported that thyroid cancer may play a part in aging, subclinical and overt thyroid cancer are more common disorders in elderly, but the exact mechanism remains unclear (34, 35).

The direct relationship between aging and thyroid cancer is currently unclear, but we speculate it may be due to the following reasons. Firstly, aging is accompanied by the shortening of cell telomeres and the accumulation of DNA damage. Telomeres are the protective structures at the end of chromosomes, and their length gradually shortens during each cell division. When telomeres are shortened to a certain extent, cells enter an aging state or apoptosis. However, in some cases, telomere shortening can lead to chromosomal instability and increase the likelihood of carcinogenesis (36). In addition, as age increases, the efficiency of DNA repair mechanisms decreases, leading to the accumulation of DNA damage. This accumulated damage may lead to genetic mutations and the occurrence of cancer (36). Secondly, the decline in immune system function is also an important characteristic of aging. Immunosenescence describes the phenomenon of the immune system’s decline in function with age, including a decrease in the efficiency of immune monitoring (37). Immune surveillance is the process by which the body recognizes and clears abnormal cells. As immune function weakens, mutated cells in the body are more likely to evade immune system monitoring, thereby increasing the risk of cancer, including thyroid cancer. In addition, aging is accompanied by an increase in chronic inflammation, which is known as “inflammatory aging” (38). Chronic low-grade inflammation may contribute to the onset and progression of cancer through multiple pathways. The inflammatory microenvironment can facilitate tumor cell proliferation, angiogenesis, and metastasis, all of which are significant factors in the development of thyroid cancer (39).

In the context of thyroid cancer, the accelerated DNA methylation age might suggest that epigenetic alterations, which accumulate with age, could influence the initiation and progression of cancer. DNA methylation can regulate gene expression by silencing or activating specific genes. Changes in DNA methylation patterns could disrupt the normal function of genes involved in cell cycle regulation, apoptosis, and DNA repair, thereby contributing to cancer development. Additionally, as thyroid cancer is more common in older individuals, accelerated epigenetic aging could reflect the cumulative effect of environmental factors, lifestyle, and genetic predisposition, all of which might interact to promote tumorigenesis. In summary, the biological implications of DNA methylation age acceleration in thyroid cancer risk could be interpreted as a signal of underlying epigenetic changes that promote a pro-cancerous environment, accelerating disease progression. Further research could investigate the specific genes and pathways influenced by these methylation changes to better understand the mechanistic link between aging and cancer susceptibility. And, The findings of this study provide a novel perspective on the relationship between aging, immune senescence, and thyroid cancer, which could have significant clinical implications. The identification of DNA methylation age acceleration and the potential epigenetic markers associated with thyroid cancer risk offers new opportunities for early detection and risk stratification. For instance, the DNA methylation signatures identified in this study could potentially be used as biomarkers to assess individuals at high risk for thyroid cancer, particularly in older populations where the disease burden is higher. Implementing such biomarkers in routine clinical practice would enable earlier interventions, personalized surveillance, and potentially even preventive strategies for high-risk individuals.

While our study provides valuable insights into the causal relationship between aging and thyroid cancer, we acknowledge several limitations that may impact the interpretation and generalizability of our findings. First, although we have made efforts to minimize the influence of unmeasured confounding and reverse causality bias, these factors remain a potential concern, as they are inherent to observational studies. The applicability of our findings may be limited to populations of European descent, as genetic variants and their effects can differ across ethnic groups. This emphasizes the need for replication studies in diverse populations to confirm whether our conclusions hold true in non-European cohorts and to ensure their broader applicability. Another limitation of our study, which is common in MR research, is the potential for unobserved horizontal pleiotropy. Horizontal pleiotropy occurs when genetic variants influence multiple traits through pathways unrelated to the exposure of interest, which could bias the estimation of causal effects. While we employed sensitivity analyses to assess and minimize the impact of pleiotropy, the possibility of residual pleiotropic effects cannot be fully excluded. Future studies with more refined instruments or advanced statistical techniques to detect and account for horizontal pleiotropy could further strengthen the robustness of our findings. In summary, while we have made several efforts to mitigate potential biases in our study, further validation in diverse populations and with improved statistical methods is necessary to confirm the causal relationship between aging and thyroid cancer. These steps will enhance the credibility and generalizability of our conclusions, making them more applicable to broader clinical contexts.

## Conclusion

In conclusion, our findings provide initial evidence suggesting a causal effect of the aging process on thyroid cancer.

## Data Availability

The datasets presented in this study can be found in online repositories. The names of the repository/repositories and accession number(s) can be found in the article/**Supplementary Material**.
